# Myo-inositol supplementation for prevention of gestational diabetes mellitus in overweight and obese pregnant women: a systematic review and meta-analysis

**DOI:** 10.1186/s13098-022-00862-5

**Published:** 2022-07-06

**Authors:** Sepideh Mashayekh-Amiri, Sakineh Mohammad-Alizadeh-Charandabi, Somaiyeh Abdolalipour, Mojgan Mirghafourvand

**Affiliations:** 1 Department of Midwifery, Faculty of Nursing & Midwifery, Tabriz University of Medical Sciences, Tabriz, Iran; 2 Menopause Andropause Research Center, Ahvaz Jundishapur University of Medical Sciences, Ahvaz, Iran; 3 Social Determinants of Health Research Center, Tabriz University of Medical sciences, Tabriz, Iran

**Keywords:** Myo-inositol supplementation, Gestational diabetes mellitus, GDM, Overweight and obese

## Abstract

**Background:**

The prevalence of gestational diabetes mellitus [GDM] and of its most important predisposing factor, i.e. overweight and obesity, have increased dramatically over the past 20 years. Therefore, the aim of this study was to systematically review the articles on the effect of myo-inositol supplementation on the prevention of GDM in pregnant women with overweight and obesity.

**Methods:**

We conducted a systematic literature search in electronic database (MEDLINE, Cochrane Library, ClinicalTrials.gov, Embase, ProQuest, PubMed, Google scholar, Scopus, Web of science and forward and backward citations) to identify all randomized controlled trials (RCTs) published until 21 December 2021. Finally, Among the 118 identified records, four studies were eligible and were included in this systematic review. The meta-analysis results were reported in the form of odds ratio (OR) to compare the incidence of GDM and pregnancy outcomes. They were also presented in the form of mean difference (MD) to compare fasting glucose (FG), 1-h and 2-h oral glucose tolerance test (OGTT) levels between the two groups. This study was registered on PROSPERO, number CRD42021290570.

**Results:**

The results showed that the incidence of GDM was significantly lower in the myo-inositol group (OR 0.32, 95% CI 0.21 to 0.48; P < 0.001; I^2^ = 0%; Moderate certainty evidence). Moreover, FG-OGTT (MD − 2.64 mg/dl, 95% CI − 4.12 to − 1.17; P < 0.001; I^2^ = 0%; Moderate certainty evidence), 1-h-OGTT (MD − 7.47 mg/dl, 95% CI − 12.24 to − 2.31; P = 0.005; I^2^ = 27%; Low certainty evidence) and 2-h-OGTT levels (MD − 10.51 mg/dl, 95% CI − 16.88 to − 4.14; P = 0.001; I^2^ = 59%; Low certainty evidence) in the myo-inositol group were significantly lower than in the control group. Regarding the pregnancy outcomes, the incidence of gestational hypertension and preterm delivery was significantly lower in the myo-inositol group. However, no between-group difference was observed in the other outcomes.

**Conclusions:**

Based on the results, myo-inositol has shown to be a new and safe preventive strategy in reducing the incidence of GDM and in regulating FG and 1-h and 2-h OGTT levels, and also in reducing the incidence of GDM complications such as preterm delivery and gestational hypertension in pregnant women with overweight and obesity.

## Background

Due to the sedentary lifestyle following urbanization and the epidemiological transition of the populations to aging in recent decades, the prevalence of type 2 diabetes mellitus (T2DM) has been increasing rapidly among younger adults worldwide [1]. Currently, many developing countries suffer from the increasing burden of T2DM and the complications associated with it.

Because of the physiological and metabolic changes that are made during pregnancy to provide the fetus with the required nutrients and oxygen, diabetic conditions develop that are similar to those of T2DM [2]. As a result, the prevalence of gestational diabetes mellitus (GDM), which has turned into one of the most common pregnancy complications and an emerging epidemic worldwide, has increased by more than 30% in some countries, including developing countries, over the past decade [3]. At present, hyperglycemia affects one in six pregnancies worldwide [4].

GDM is defined as a disorder of glucose tolerance and carbohydrate intolerance, which first begins or is diagnosed during pregnancy and is associated with symptoms such as high blood glucose, increased insulin resistance, decreased insulin sensitivity, and increased insulin requirements [5]. At the global level, based on the estimate made by the International Diabetes Federation [IDF], GDM affects 14% of pregnancies [6]. The results of a systematic review study in Asia showed an 11.5% prevalence of this metabolic disorder [7].

Gestational diabetes mellitus (GDM) is accompanied by a wide range of undesired side effects for both mother and fetus including macrosomia, preeclampsia, shoulder dystocia, and neonatal hyper bilirubinemia [8]. Moreover, the increase in the risk of metabolic diseases such as T2DM in mothers and their babies after pregnancy leads to a great economic burden on the government, the community, and the families [9]. A wide range of GDM complications for mother and infant highlights the need for diagnosing it and screening for it. Many factors are proposed as predisposing factors for GDM. Risk factors including obesity and overweight, advanced maternal age, history of two or more pregnancies, family history of T2DM, previous history of GDM, polycystic ovarian syndrome (PCOS), persistent glucosuria, recurrent miscarriages, previous history of macrosomia (birth weight ≥ 4000 g), history of stillbirth, history of chronic hypertension, gestational hypertension and maternal smoking increase the risk of the development of GDM in pregnant women [10, 11].

Among the risk factors, overweight and obesity are very important in pregnant women. In fact, the risk of GDM in pregnant women who are overweight and obese, as well as in women with morbid obesity, is 2, 4, and 8 times higher, respectively [12]. On the other hand, the chances of pregnancy complications, such as preeclampsia and fetal growth restriction, are higher in pregnant women with overweight and obesity [13, 14]. The results from some systematic review studies indicated a positive correlation between GDM and increased body mass index (BMI) in pregnant women [15, 16]. The growing prevalence of obesity worldwide and the consequent increase in the incidence of GDM emphasize the importance of using preventive strategies to prevent unwanted consequences of obesity and hyperglycemia during pregnancy [17].

There are several strategies for preventing GDM including diet and exercise intervention and dietary supplement interventions such as vitamin D [18] probiotics [19] and fish oil [20] are used to prevent GDM. The results of an overview of Cochrane Reviews about interventions to prevent from developing GDM, showed that vitamin D, had a possible benefit effect while vitamin D with calcium supplementation and probiotics had an unclear effect and fish oil supplementation in pregnancy had no effect on the risk of developing GDM [21]. Currently, the first recommended step in preventing GDM is diet correction and physical activity [21].

In this regard, the results of a systematic review showed that physical activity alone did not have a significant effect on the overall incidence of GDM in pregnant women with overweight and obesity [22]. Consequently, available pharmacological or non-pharmacological techniques have not been accompanied by promising or long-term consequences for mothers and their infants [23]. Due to the lack of safe and effective strategies to prevent GDM, it is essential to identify and use new supplements [24].

Myo-inositol can be used as a new, effective, approved, and simple supplement for GDM prevention by controlling maternal blood glucose levels without harming the mother and fetus [25]. In recent years, a broad range of studies has been conducted on the efficacy and safety of myo-inositol for the prevention of GDM [26–28]. However, a recent Cochrane systematic review showed the poor quality of the related evidence [29].

Myo-inositol is a vitamin-like cyclic polyols that belongs to the vitamin B family. However, defining inositol as a vitamin is not entirely correct since it is produced in sufficient quantities by the human liver, kidneys, and brain and is naturally present in fresh fruits and vegetables, grains, legumes, and nuts [30, 31]. Inositol is described as a second messenger and an insulin sensitizer that improves glucose homeostasis and plays an important role in glucose regulation [32]. Recent studies have shown that myo-inositol supplementation has insulin-sensitizing effects, reduces insulin resistance following pregnancy, and increases BMI. Its beneficial effects in reducing GDM rate in normal-weight pregnant women have been shown in several systematic reviews [25, 33].

Given the insulin resistance decreases by using myo-inositol and increases in pregnant women affected by obesity, there is no standard practical and safe treatment for it. There are also concerns about the increasing prevalence of GDM and obesity. Consequently, this research reviewed the clinical trials in which myo-inositol supplementation was used to prevent GDM in pregnant women with overweight and obesity. No systematic review concerning the effect of myo-inositol supplementation on GDM in pregnant women with overweight and obesity was found in the search for research articles.

## Methods

### Inclusion and exclusion criteria

All the published and unpublished clinical trials and conference abstracts in English and Persian that investigated the efficacy of myo-inositol supplementation with different doses for GDM prevention in pregnant women with overweight and obesity were included in this research. Clinical trials on women with a history of GDM, a history of pre-gestational diabetes, glycosuria in the first trimester of pregnancy, and treatment with corticosteroids were excluded. Studies on the combined effect of myo-inositol with other supplements, except for folic acid which must be prescribed during pregnancy, were not included in the study. Moreover, cross-sectional and quasi-experimental clinical trials were excluded.

### Types of participants

Pregnant women with overweight and obesity (BMI ≥ 25) were included in this.

### Types of interventions

The intervention included receiving different doses of myo-inositol in combination with folic acid. The control group included no treatment, or received a placebo or folic acid as the placebo.

### Types of outcome measures

The primary outcomes of the study were incidence of GDM, FG level, 1-h and 2-h OGTT levels in the second trimester of pregnancy. The secondary outcomes included pregnancy outcomes: gestational hypertension, caesarean section, preterm delivery, macrosomia, shoulder dystocia, neonatal hypoglycaemia as well as the need for transfer to the Neonatal Intensive Care Unit (NICU).

### Search methods for identification of studies

We conducted a systematic literature search in electronic database (MEDLINE, Cochrane Library, ClinicalTrials.gov, Embase, ProQuest, PubMed, Google scholar, Scopus, Web of science and forward and backward citations) to find all randomized controlled trials (RCTs) that using myo-inositol supplementation (Myo-inositol supplement plus 200 µg of folic acid) for prevention of GDM was compared with the control group (placebo, 200 mcg of folic acid) in pregnant women with overweight and obesity, with the keywords (Myo-inositol supplementation, gestational diabetes mellitus, GDM, overweight, obese, randomized controlled trials, meta-analysis) from the database inception until December 21, 2021. The references used in these studies were manually searched to identify more relevant studies not captured by electronic searches. As an example, the strategic search for the PubMed database was as follows:

("Myo-Inositol-1-Phosphate Synthase"[MeSH Terms] OR "Inositol"[Text Word] OR "Myoinositol"[Text Word] OR "Myo-inositol"[Text Word]) AND ("diabetes, gestational"[MeSH Terms] OR "Pregnancy-Induced Diabetes"[Text Word] OR "Gestational Diabetes Mellitus"[Text Word] OR "GDM"[Text Word]) AND ("Overweight"[MeSH Terms] OR "Obesity"[MeSH Terms]).

### Data collection and analysis

#### Selection of studies

Two review authors (SMA and SA) independently reviewed the titles and abstracts of the extracted literature for eligibility criteria. In the absence of sufficient information and inference in the titles and abstracts of the studies, their full texts were reviewed for inclusion. Disagreements between the two authors concerning the eligibility of the studies were resolved through discussion. If they could not be resolved, a third person (MM) was consulted. The study flow diagram shows the number of identified records and the number of included and excluded studies.

#### Data extraction and management

To review the eligible studies, two review authors (SMA and SA) independently extracted specifications of the studies using the data extraction form and resolved any disagreements that arose by discussion. The data independently extracted by the authors included the name of the first author, year of the study, numbers of participants in the study groups, intervention details, BMI values, inclusion and exclusion criteria, outcomes assessment, and results. In this regard, the corresponding authors of the studies with incomplete or inadequate data in each stage were contacted (Table 1).

**Table 1:** Characteristics of included studies

Authors	Location	Type of the study	Inclusion criteria	Population		Intervention	Results
				MI^b^	Placebo		
Danna, 2015	Italy	Randomized controlled open-label study	BMI > 30 kg/m^2^ Obese first-trimester fasting plasma glucose < 126 mg/dl	110	110	2 g MI + 200 mcg folic acid vs. 200 mcg folic acid twice/day	The GDM rate was significantly reduced in the MI group compared with the control group, 14.0% compared with 33.6%, respectively [P < 0.001; odds ratio 0.34, 95% confidence interval 0.17– 0.68]
Santamaria, 2016	Italy	Randomized controlled open-label study	BMI ≥ 25 kg/m^2^ and < 30 kg/m^2^ Overweight first-trimester fasting plasma glucose < 126 mg/dl and/or random glycaemia < 200 mg/dl	110	110	2 g MI + 200 mcg folic acid vs. 200 mcg folic acid twice/day	The incidence of GDM was significantly lower in the MI group compared to the placebo group [11.6% versus 27.4%, respectively, p¼0.004]. MI treatment was associated with a 67% risk reduction of developing GDM [OR 0.33; 95% CI 0.15, 0.70]
Vitale, 2020	Italy	Randomized controlled open-label study	BMI > 25 kg/m^2^ and < 30 kg/m^2^ Overweight first-trimester fasting plasma glucose < 126 mg/dl and/or random glycaemia < 200 mg/dl	110	110	2 g MI + 200 mcg folic acid vs. 200 mcg folic acid twice/day	The global incidence of GDM was significantly reduced in the MI group [n = 9, 8.2%] compared with the placebo group [n = 24, 21.2%] [p = 0.006]
Esmailzadeh,2022	Iran	Randomized controlled double-blind study	BMI ≥ 25 kg/m^2^ and < 30 kg/m^2^ Overweight first-trimester fasting plasma glucose < 126 mg/dl and/or random glycaemia < 200 mg/dl	30	30	2 g MI + 200 mcg folic acid vs. 400 mcg folic acid daily	The incidence of gestational diabetes in MI group was noticeably minimized compared with that of the control group [RR 0.29, 95% CI 0.09–0.94, p = 0.037]

^a^*BMI* Body mass index, ^b^*MI* Myoinositol, ^b^*GDM* Gestational diabetes mellitus

#### Assessment of risk of bias in included studies

Two review authors (SMA and SA) independently examined the risk of bias for all of the included studies using the criteria listed in the Cochrane Handbook. The risk of bias was assessed using the Cochrane risk of bias (RoB, version 2.0) tool. This instrument is comprised of five domains. The risk of bias of each item for the included studies was classified under the topics. In the next step, the judgments were matched, any disagreement was resolved by consulting with a third person, and the final result was obtained. Finally, a study in which all five areas were at low risk was regarded with an overall low risk for bias. A study in which at least one area was with some risk concerns was regarded with some risk concerns for bias. A study in which at least one area was at high risk or more than one area were with some concerns was regarded with high risk for bias [34].

#### Assessing the quality of the body of evidence using the GRADE approach

The quality of evidence provided in the included studies was examined in terms of the main outcomes of the five domains (Risk of bias, Imprecision, Inconsistency, Indirectness and Publication bias) in the GRADE (The Grades of Recommendation, Assessment, Development & Evaluation Working Group) approach [35].

#### Statistical method

The data were analyzed using Review Manager Version 5.3 (The Nordic Cochrane Centre, Cochrane Collaboration, 2014, Copenhagen, Denmark). Data on the incidence of GDM, pregnancy outcomes and FG, and 1-h and 2-h OGTT were extracted for the intervention and control groups of each study. The meta-analysis results were reported in the form of OR to compare the incidence of GDM and pregnancy outcomes between the two groups. They were also presented in the form of MD to compare FG, 1-h and 2-h OGTT levels between the two groups. Heterogeneity was investigated using the I^2^, Tau^2^, and Chi^2^. Heterogeneity was considered if I_2_ > 30 and Tau^2^ > 0 or P value less than 0.10 in Chi^2^ test [36]. If significant heterogeneity was observed, subgroup analyses and sensitivity analyses would be used [37].

## Results

### Description of studies

**Fig. 1:**
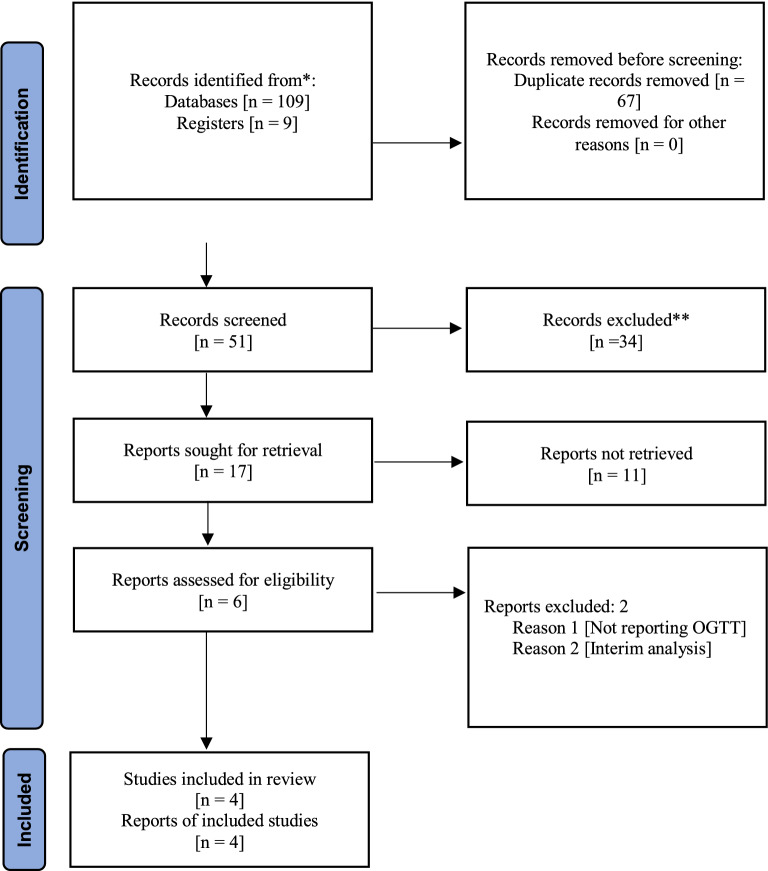
PRISMA flow diagram [2020] of screening, selection process and inclusion study

In the initial search of the database, 118 articles were found (109 articles from the databases and nine articles from the registries). However, 67 articles were identified and removed due to duplication using Endnote software. Of the remaining 51 articles, 34 were removed in the first level of screening (the title and abstract review). In the second level of screening (the full-text review), 13 of the remaining 17 articles were excluded due to ineligibility. Finally, four articles entered the meta-analysis stage of the research (Fig. 1).

### Characteristics of included studies

#### Study design

These four eligible trials were conducted in parallel design (three open-label studies, one double-blind study). The participants in three studies were divided into the intervention group [receiving INOFULIC^®^, which was produced in Italy, twice a day] and the control group. Moreover, the participants in the research by Esmaielzadeh et al. were divided into the intervention group (receiving the supplement once a day) and the control group [27]

#### Setting

Three trials were conducted in Italy and one trial in Iran.

#### Participants

All trials were conducted in pregnant women with overweight and obesity (BMI ≥ 25).

#### Gestational age at trial entry

The gestational age at enrollment and the beginning of the intervention in pregnant women was 12–13-weeks in three studies [26, 28, 38] and 12–14-weeks in the other study [27].

#### Body mass index (BMI)

Three of the four studies have been conducted on pregnant women with overweight [27, 28, 38], and one on pregnant women with obesity [26].

#### Duration of intervention and comparison

The duration of the intervention in two studies [26, 28] was until delivery, in one study [38] it was until three weeks after delivery, and in the other study it was from 12-14-weeks of gestation for 10 weeks [27].

#### Outcome measures

In all four studies, FG, and 1-h and 2-h OGTT levels in the second trimester of pregnancy were measured at 24–28-week of gestation based on the diagnostic criteria by the American Diabetes Association (ADA), i.e. OGTT with 75 g of oral glucose. According to these criteria, if one of these values exceeded the determined limit, it would be indicative of GDM. Pregnancy outcomes, such as gestational hypertension, were reported in all studies [26–28, 38], cesarean delivery, preterm delivery, macrosomia, shoulder dystocia and admission to the NICU in three studies [26–28] and neonatal hypoglycemia [26, 28] in two studies. Given the outcomes assessment, quantitative variables, such as FG, and 1-h and 2-h OGTT levels were reported in the form of (mean ± SD) separately for the groups. The qualitative variables, such as GDM rate, as well as pregnancy outcomes, such as gestational hypertension, cesarean section, preterm delivery, macrosomia, shoulder dystocia, neonatal hypoglycemia and admission to NICU, were reported in percentage and frequency in all the studies.

#### Risk of bias in included studies

**Fig. 2:**
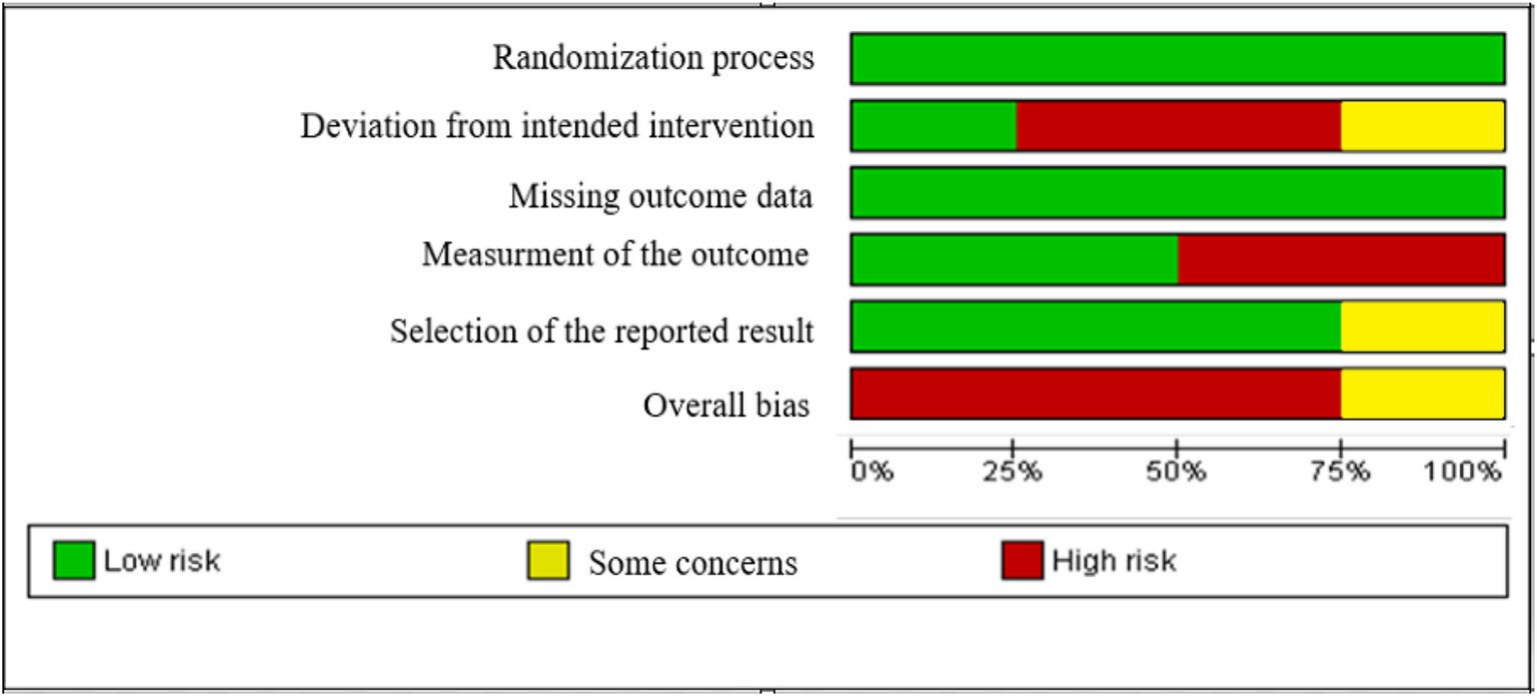
Risk of bias graph: review authors’ judgements about each risk of bias item presented as percentages across all included studies

**Fig. 3:**
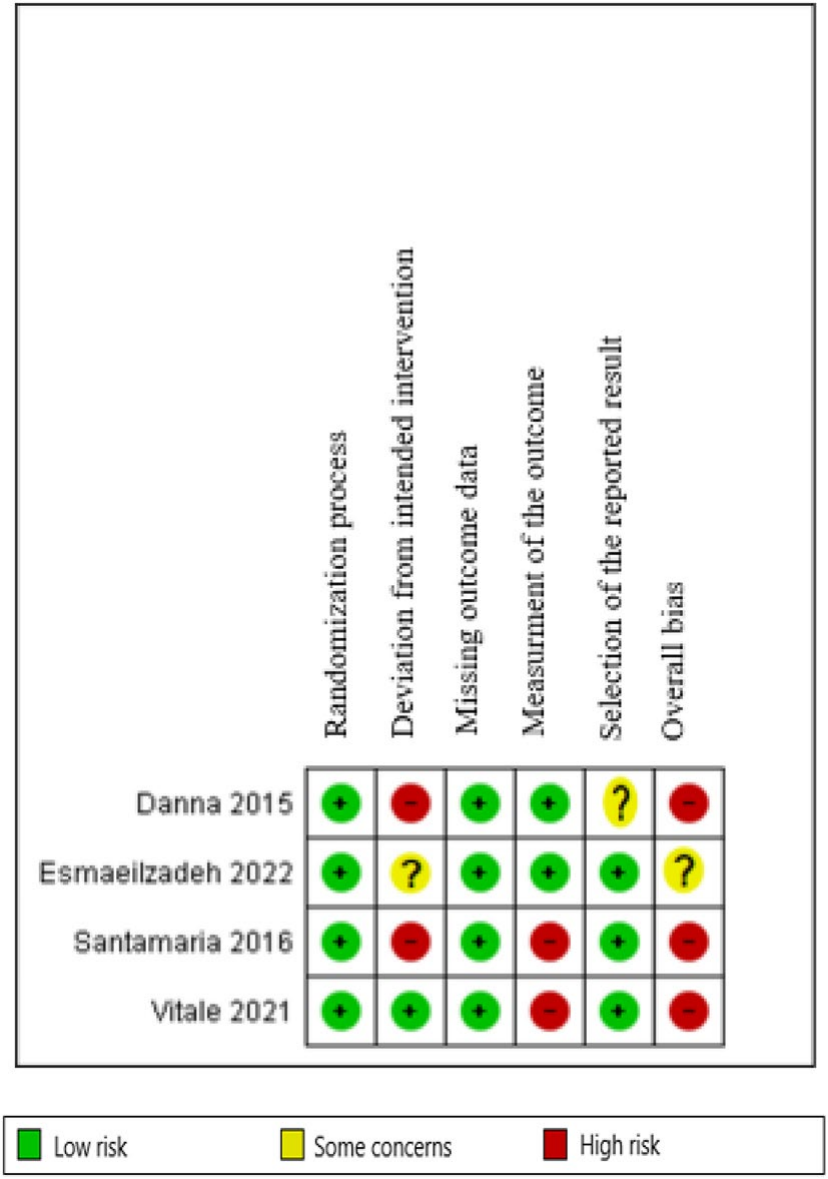
Risk of bias summary: review authors’ judgements about each risk of bias item for each included study

Three of the four studies had a high risk for bias and one study [27], had some concerns in this regard. The randomization process was at low risk in all studies [26–28, 38]. The second domain, i.e. deviation from the intended intervention, was at high risk in two studies [26, 28], with some concerns in one [27], and at low risk in one [38]. The missing outcome data were at low risk in all studies [26–28, 38]. The outcomes assessment was at low risk in two studies [26, 27], and at high risk in two [28, 38]. Finally, the selection of reported results was at low risk in three studies and with some concerns in one [26] (Figs. 2, 3).

#### Meta-analysis of included studies

##### Gestational diabetes mellitus [GDM]

**Fig. 4:**
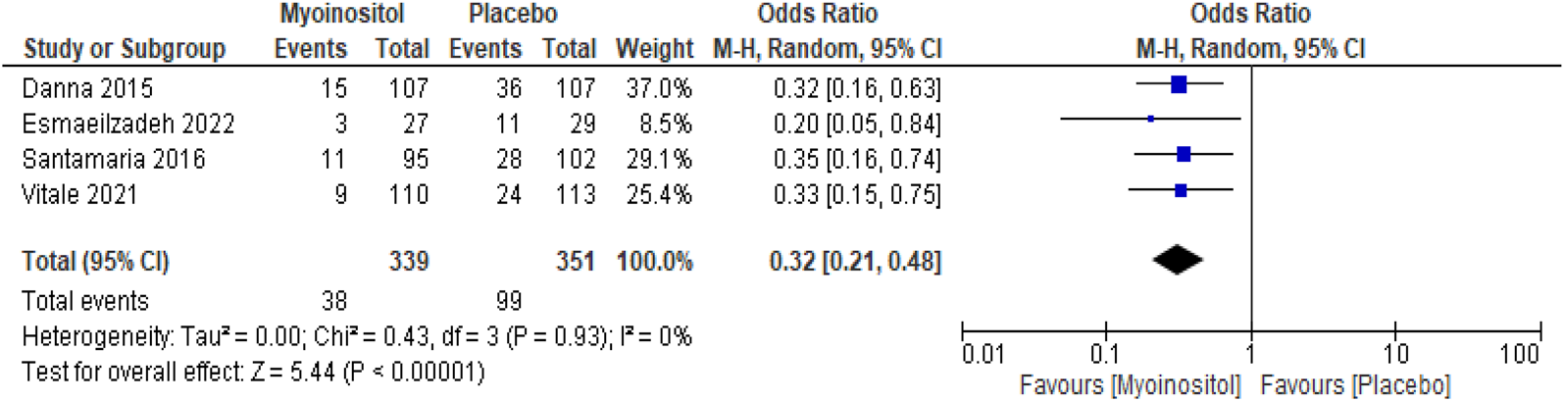
Myoinositol vs. Placebo, outcome: Gestational diabetes mellitus

The overall results of the meta-analysis of the four trials with 690 participants showed that myo-inositol supplementation was accompanied by a significant reduction (OR 0.32, 95% CI 0.21 to 0.48; P < 0.001; I^2^ = 0%; Moderate certainty evidence) in the incidence of GDM compared to the control group (Fig. 4).

##### FG-OGTT

**Fig. 5:**
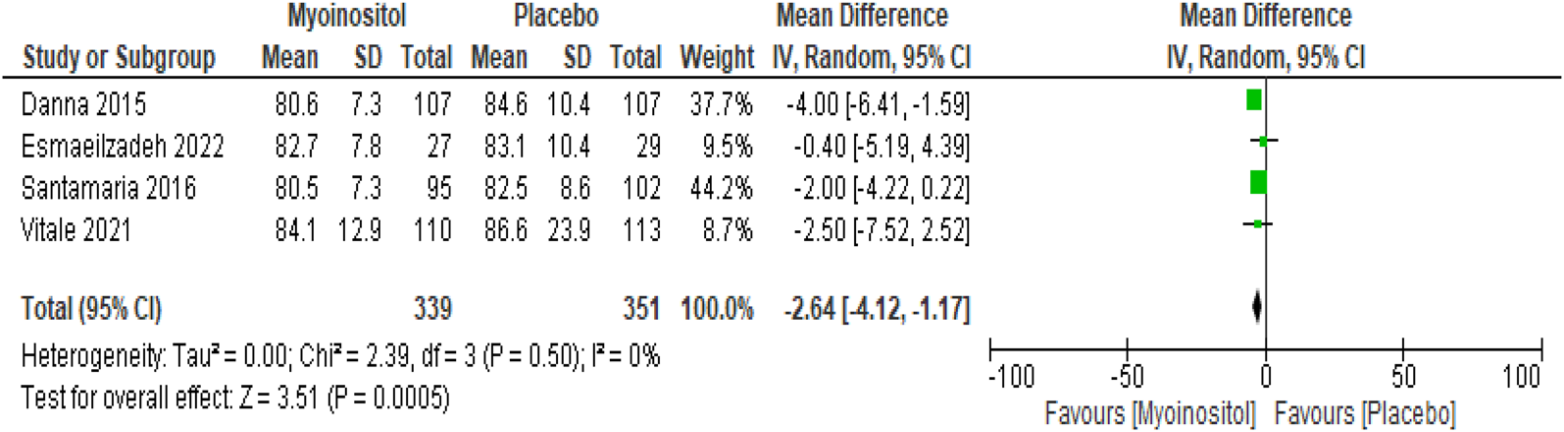
Myoinositol vs. Placebo, outcome: FG-OGTT

The overall results of the meta-analysis of the four trials with 690 participants showed that myo-inositol supplementation was accompanied by a significant reduction (MD − 2.64 mg/dl, 95% CI − 4.12 to − 1.17; P < 0.001; I^2^ = 0%; Moderate certainty evidence) in the FG-OGTT levels compared to the control group (Fig. 5).

##### hour-OGTT

**Fig. 6:**
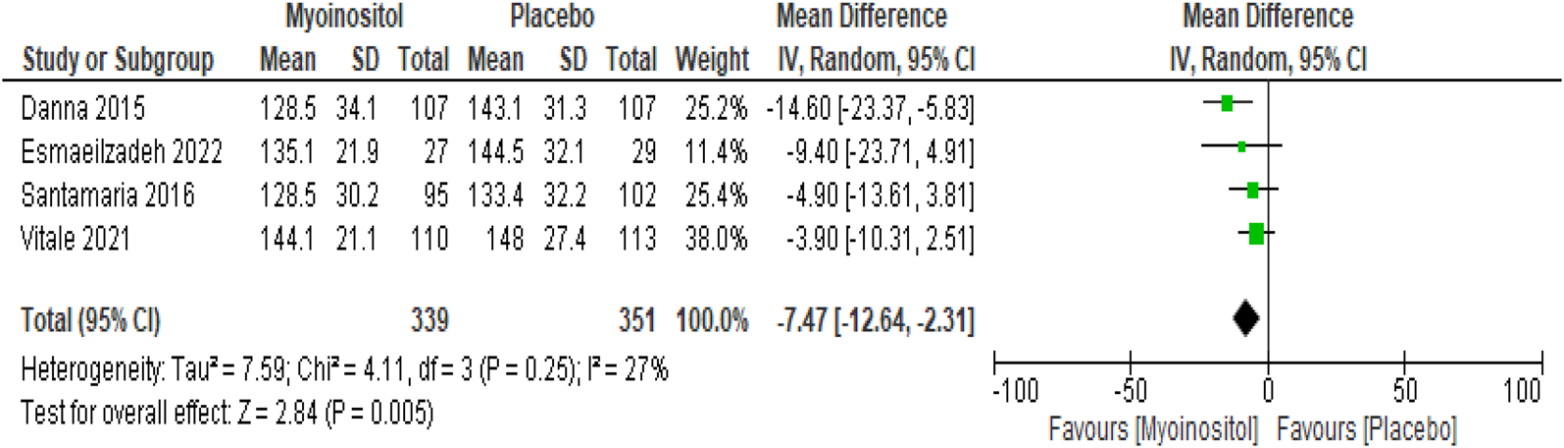
Myoinositol vs. Placebo, outcome: 1 h-OGTT

The overall results of the meta-analysis of the four trials with 690 participants showed that myo-inositol supplementation was accompanied by a significant reduction (MD − 7.47 mg/dl, 95% CI − 12.24 to − 2.31; P = 0.005; I^2^ = 27%; Low certainty evidence) in the 1 h-OGTT levels compared to the control group (Fig. 6).

##### hour-OGTT

**Fig. 7:**
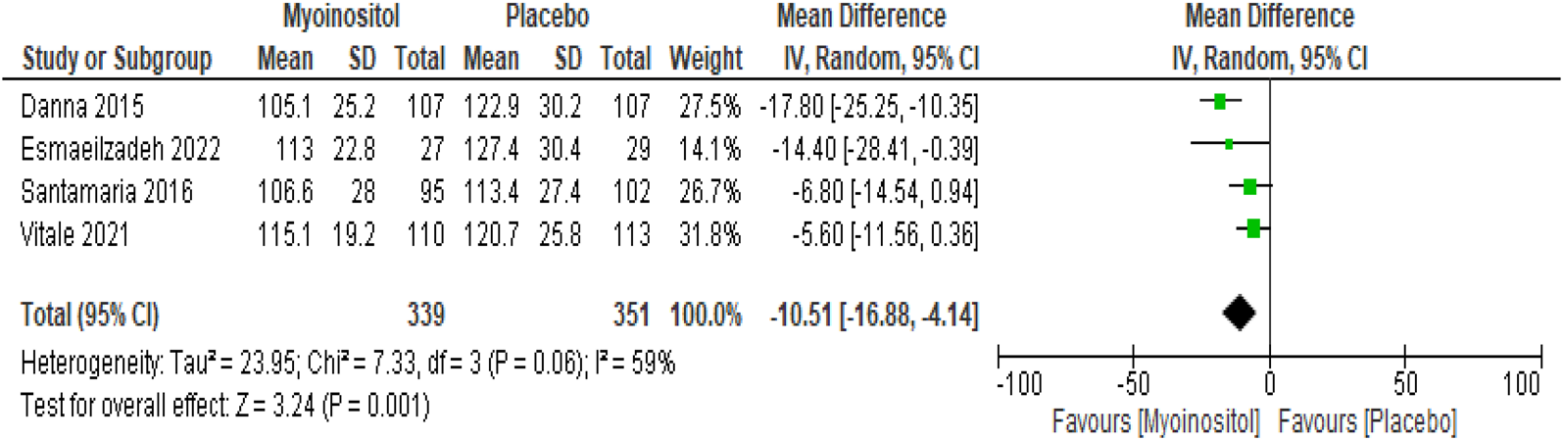
Myoinositol vs. Placebo, outcome: 2 h-OGTT

The overall results of the meta-analysis of the four trials with 690 participants showed that myo-inositol supplementation was accompanied by a significant reduction (MD -10.51 mg/dl, 95% CI − 16.88 to − 4.14; P = 0.001; I^2^ = 59%; Low certainty evidence) in the 2 h-OGTT levels compared to the control group (Fig. 7).

##### Gestational hypertension

**Fig. 8:**
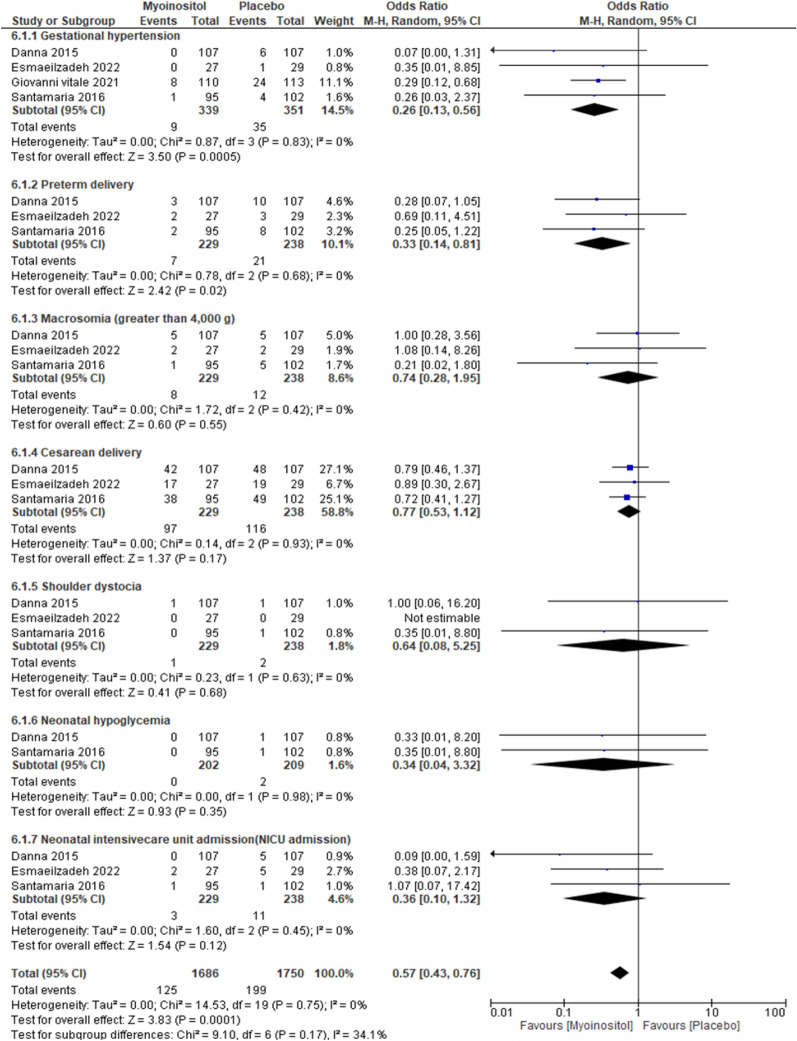
Myoinositol vs. Placebo, Pregnancy outcomes

The overall results of the meta-analysis of the four trials with 690 participants showed that myo-inositol supplementation was accompanied by a significant reduction (OR 0.26, 95% CI 0.13 to 0.56; P < 0.001; I^2^ = 0%; Moderate certainty evidence) in the incidence of gestational hypertension compared to the control group (Fig. 8).

##### Caesarian section

The overall results of the meta-analysis of the three trials with 467 participants showed that myo-inositol supplementation wasn’t accompanied by a significant reduction (OR 0.77, 95% CI 0.53 to 1.12; P = 0.17; I^2^ = 0%; Low certainty evidence) in the caesarian section rate compared to the control group (Fig. 8).

##### Preterm delivery

The overall results of the meta-analysis of the three trials with 467 participants showed that myo-inositol supplementation was accompanied by a significant reduction (OR 0.33, 95% CI 0.14 to 0.81; P = 0.02; I^2^ = 0%; Low certainty evidence) in the incidence of preterm delivery compared to the control group (Fig. 8).

##### Macrosomia

The overall results of the meta-analysis of the three trials with 467 participants showed that myo-inositol supplementation wasn’t accompanied by a significant reduction (OR 0.74, 95% CI 0.28 to 1.95; P = 0.55; I^2^ = 0%; Low certainty evidence) in the macrosomia rate compared to the control group (Fig. 8).

##### Shoulder dystocia

The overall results of the meta-analysis of the three trials with 467 participants showed that myo-inositol supplementation wasn’t accompanied by a significant reduction (OR 0.64, 95% CI 0.08 to 5.25; P = 0.68; I^2^ = 0%; Very low certainty evidence) in the shoulder dystocia rate compared to the control group (Fig. 8).

##### Neonatal hypoglycemia

The overall results of the meta-analysis of the three trials with 467 participants showed that myo-inositol supplementation wasn’t accompanied by a significant reduction (OR 0.34, 95% CI 0.04 to 3.32; P = 0.35; I^2^ = 0%; Very low certainty evidence) in the neonatal hypoglycemia rate compared to the control group (Fig. 8).

##### NICU admission

The overall results of the meta-analysis of the three trials with 467 participants showed that myo-inositol supplementation wasn’t accompanied by a significant reduction (OR 0.36, 95% CI 0.10 to 1.32; P = 0.12; I^2^ = 11.8%; Low certainty evidence) in the NICU admission compared to the control group (Fig. 8).

##### Overall quality of evidence

The overall quality of evidence was rated as low for all the outcomes evaluated (Table 2). The majority of studies were at high risk of bias as well as at high risk due to high inconsistency and small sample size (i.e. small number of patients and events) (Table 3).

**Table 2:** Use of the Myoinositol versus Placebo

No. of studies	Design	Risk of bias	Inconsistency	Indirectness	Imprecision	Other considerations	Use of the MI	No Use of the MI	Pooled effect Relative^a^ [95% CI]	Final judgment
Gestational diabetes rate [GDM Rate]										
4	RCTs	Serious^b^	No serious	No serious	No serious	No serious	38/339	99/351	OR 0.32 [0.21 to 0.]48]	⨁⨁⨁Ο MODERATE
FG-OGTT										
4	RCTs	Serious^b^	No serious	No serious	No serious	No serious	339	351	MD 2.64 lower [4.12 to 1.17 lower]	⨁⨁⨁Ο MODERATE
1 h-OGTT										
4	RCTs	Serious^b^	No serious	No serious	No serious	No serious	339	351	MD 7.47 lower [12.24 to 2.31 lower]	⨁⨁ΟΟ LOW
2 h-OGTT										
4	RCTs	Serious^b^	Serious^2^	No serious	No serious	No serious	339	351	MD 10.51 lower [16.88 to 4.14 lower]	⨁⨁ΟΟ LOW
Gestational hypertension										
4	RCTs	Serious^b^	No serious	No serious	No serious	No serious	9/339	35/351	OR 0.26 [0.13 to 0.56]	⨁⨁⨁Ο MODERATE
Preterm labor										
3	RCTs	Serious^b^	No serious	No serious	Serious^d^	No serious	7/229	21/238	OR 0.33 [0.14 to 0.81]	⨁⨁ΟΟ LOW
Cesarean section rate										
3	RCTs	Serious^b^	No serious	No serious	Serious^d^	No serious	97/229	116/238	OR 0.77 [0.53 to 1.12]	⨁⨁ΟΟ LOW
Macrosomia										
3	RCTs	Serious^b^	No serious	No serious	Serious^d^	No serious	8/229	12/238	OR 0.74 [0.28 to 1.95]	⨁⨁ΟΟ LOW
Neonatal hypoglycemia										
2	RCTs	Serious^b^	No serious	No serious	Very serious^d^	No serious	0/202	2/209	OR 0.34 [0.04 to 3.32]	⨁ΟΟΟ VERY LOW
NICU admission										
3	RCTs	Serious^b^	No serious	No serious	Serious^d^	No serious	3/229	11/238	OR 0.36 [0.10 to 1.32]	⨁⨁ΟΟ LOW
Shoulder dystocia										
3	RCTs	Serious^b^	No serious	No serious	Very serious^d^	No serious	1/229	2/238	OR 0.64 [0.08 to 5.25]	⨁ΟΟΟ VERY LOW

GRADE Working Group grades of evidence

High quality: We are very confident that the true effect lies close to that of the estimate of the effect

Moderate quality: We are moderately confident in the effect estimate: The true effect is likely to be close to the estimate of the effect, but there is a possibility that it is substantially different

Low quality: Our confidence in the effect estimate is limited: The true effect may be substantially different from the estimate of the effect

Very low quality: We have very little confidence in the effect estimate: The true effect is likely to be substantially different from the estimate of effect

*CI* confidence interval, *OR* odds ratio, *OGTT* oral glucose tolerance test, *NICU* neonatal intensive care unit admission

^a^The risk in the intervention group [and its 95% confidence interval] is based on the assumed risk in the comparison group and the relative effect of the intervention [and its 95% CI]

^b^Studies at high risk of bias

^c^Severe unexplained heterogeneity

^d^Wide confidence interval [CI] crossing the line of no effect

^e^Small sample size and/or few events

**Table 3:** Summary of findings for the main comparison. Myoinositol supplementation compared to placebo for gestational diabetes mellitus and health outcomes

Summary of findings
Myoinositol compared to Placebo for gestational diabetes prevention
Patient or population: overweight and obese Pregnant women at increased risk of gestational diabetes
Setting: trials were carried from 1980s to 2021 in countries from Italy, Iran
Intervention: Myoinositol
Comparison: Placebo

Outcomes	Anticipated absolute effects^a^ [95% CI]		Relative effect [95% CI]	No of participants [studies]	Quality of the evidence [GRADE]
	Risk with Placebo	Risk with Myoinositol			
Gestational diabetes rate [GDM Rate]	282 per 1000	112 per 1000 [66 to 141]	OR 0.32 [0.21 to 0.48]	690 [4 RCTs]	⨁⨁⨁Ο MODERATE
FG-OGTT	The mean of FG in the control groups was 84.7	The mean of FG in the intervention groups was 2.64 lower [4.12 to 1.17 lower]		690 [4 RCTs]	⨁⨁⨁Ο MODERATE
1 h-OGTT	The mean of 1 hour-OGTT in the control groups was 142.3	The mean of 1 hour-OGTT in the intervention groups was 7.47 lower [12.24 to 2.31 lower]		690 [4 RCTs]	⨁⨁ΟΟ LOW
2 h-OGTT	The mean of 2 h-OGTT in the control groups was 121.1	The mean of 2 h-OGTT in the intervention groups was 10.51 lower [16.88 to 4.14 lower]		690 [4 RCTs]	⨁⨁ΟΟ LOW
Gestational hypertension	27 per 1000	100 per 1000 [4 to 15]	OR 0.26 [0.13 to 0.56]	690 [4 RCTs]	⨁⨁⨁Ο MODERATE
Preterm delivery	31 per 1000	88 per 1000 [4 to 25]	OR 0.33 [0.14 to 0.81]	467 [3 RCTs]	⨁⨁ΟΟ LOW
Cesarean section rate [CS rate]	424 per 1000	487 per 1000 [225 to 475]	OR 0.77 [0.53 to 1.12]	467 [3 RCTs]	⨁⨁ΟΟ LOW
Macrosomia	35 per 1000	50 per 1000 [10 to 69]	OR 0.74 [0.28 to 1.95]	467 [3 RCTs]	⨁⨁ΟΟ LOW
Neonatal hypoglycemia	0 per 1000	10 per 1000 [0]	OR 0.34 [0.04 to 3.32]	411 [2 RCTs]	⨁ΟΟΟ VERY LOW
NICU admission	13 per 1000	46 per 1000 [1 to 17]	OR 0.36 [0.10 to 1.32]	467 [3 RCTs]	⨁⨁ΟΟ LOW
Shoulder dystocia	4 per 1000	8 per 1000 [0 to 21]	OR 0.64 [0.08 to 5.25]	467 [3 RCTs]	⨁ΟΟΟ VERY LOW

GRADE Working Group grades of evidence

High quality: We are very confident that the true effect lies close to that of the estimate of the effect

Moderate quality: We are moderately confident in the effect estimate: The true effect is likely to be close to the estimate of the effect, but there is a possibility that it is substantially different

Low quality: Our confidence in the effect estimate is limited: The true effect may be substantially different from the estimate of the effect

Very low quality: We have very little confidence in the effect estimate: The true effect is likely to be substantially different from the estimate of effect

*CI* confidence interval, *OR* odds ratio, *OGTT* oral glucose tolerance test *NICU* neonatal intensive care unit admission

^a^The risk in the intervention group [and its 95% confidence interval] is based on the assumed risk in the comparison group and the relative effect of the intervention [and its 95% CI]

## Discussion

To our knowledge, and according to the database searches conducted by the researcher, the present study is the first meta-analysis of the effect of myo-inositol supplementation on preventing GDM in pregnant women with overweight and obesity. The results showed that the incidence of GDM in the myo-inositol supplementation group was significantly lower than in the control. Moreover, the FG, and 1-h, and 2-h OGTT levels in the second trimester of pregnancy were significantly lower in the myo-inositol supplementation group than in the control. Given the pregnancy outcomes, the incidence of gestational hypertension and preterm delivery was significantly lower in the myo-inositol supplementation group compared to the control. However, there was no significant between-group difference in the other outcomes such as cesarean section and macrosomia.

Nowadays, GDM has almost turned into a worldwide epidemic and can be considered a short-term metabolic syndrome that is accompanied by hyperglycemia and oxidative stress-related inflammation, which may alter intracellular signaling pathways including insulin signaling pathways [39]. This will result in such consequences as insulin resistance, and decreased insulin gene expression causing reduced insulin secretion by beta-pancreatic cells [40–42]. A wide range of short-term and long-term complications of GDM for both mother and fetus emphasizes the importance of identifying the risk factors for this metabolic disorder. One of the most common risk factors for GDM is overweight and obesity [43]. The issue of obesity and overweight in pregnancy is a public health concern due to the rapid increase in their prevalence among women of childbearing age, which can have such adverse consequences for both mother and fetus as GDM and gestational hypertension [44].

The concerning issue with regards to overweight and obesity is insulin resistance, which intensifies with increasing body fat. Insulin resistance increases further due to endocrine activity induced by adipokines produced by visceral adipose tissue in pregnant women with overweight and obesity [45]. On the other hand, pregnancy is physiologically characterized by hyperinsulinemia and insulin resistance. Moreover, insulin sensitivity during the third trimester of pregnancy decreases by 50%–70% compared to the pre-pregnancy period. Therefore, pregnancy leads to intensified insulin resistance in pregnant women with overweight and obesity [46].

As a result, preventive approaches are preferred over treatment. Recently, some supplements including myo-inositol are emerging as a new alternative. The role of myo-inositol in the intracellular transmission of insulin’s metabolic signal was first identified by Larner et al. [47]. Since then, more researchers have studied the effects of myo-inositol and its role in increasing insulin sensitivity in such diseases as PCOS, GDM, T2DM and metabolic syndrome in women during the postmenopausal period [48–50].

The biochemical mechanism by which myo-inositol improves the metabolic status of women with GDM and other insulin-resistant conditions is not known. However, there are hypotheses regarding this mechanism. One of them suggests the direct intracellular effect of myo-inositol on the activation of acetyl-CoA carboxylase (ACC), which stimulates lipogenesis [51]. The effect of myo-inositol, as an insulin-sensitizing agent, is mainly due to its effects on increasing glycogen synthesis and glucose uptake in peripheral tissues. In addition, myo-inositol, as a second messenger of insulin, may be intracellularly deficient in obese women with PCOS [52].

Moreover, urinary excretion of inositol increases in women with GDM in the first trimester of pregnancy. A randomized clinical trial on 84 pregnant women with GDM between 24 and 28-weeks of gestation investigated the effect of myo-inositol supplementation on insulin resistance parameters using the insulin resistance index (HOMA-IR) and adiponectin circulating levels. The results showed a significant reduction in HOMA-IR values, FG, and insulin levels in the myo-inositol supplementation group compared to the control [53].

The results of two preliminary systematic reviews in 2015 concerning the effect of myo-inositol supplementation on reducing the incidence of GDM (Zheng’s meta-analysis of five trials comprising 513 pregnant women with GDM) [54], and Crawford's study on four trials comprising 567 pregnant women [29], that used different myo-inositol doses and used it alone or in combination with other materials were consistent with the findings of the present study. In addition to addressing the limitations of the two previous systematic reviews, a systematic review (Guo et al.) of four clinical trials comprising 586 women with GDM risk factors [25], showed that the incidence of GDM was significantly lower in the myo-inositol supplementation group than in the control. Moreover, the FG, 1-h and 2-h blood glucose levels were significantly lower in the myo-inositol group than in the control, which confirm our findings. On the other hand, the results from the meta-analysis of five clinical trials on 927 pregnant women (Xu Jiang et al.) [24], showed that despite the reduced incidence of GDM in the myo-inositol group, there was no significant between-group difference in 2-h OGTT levels, which can be due to high heterogeneity of one of the studies in which the lower dose of myo-inositol/D-chiro-inositol caused the non-significant results. Moreover, the results of a meta-analysis (Vitagliano et al.) of five studies comprising 965 pregnant women with risk factors for GDM [33], showed that myo-inositol and/or D-chiro-inositol supplementation was able to significantly reduce the incidence of GDM and FG, based on the OGTT. However, there was no between-group difference in 1-h and 2-h OGTT levels. After the analysis of the subgroups (using 2 g of myo-inositol twice a day and 1100 g/day of myo-inositol/d-chiro-inositol), a significant reduction was observed in the incidence of GDM, FG, 1-h, and 2-h OGTT levels in the myo-inositol group (2 g twice a day) compared to the control. This difference indicates the beneficial effects of higher doses of myo-inositol (4 g/day) [33]. It is worth noting that the aforementioned studies were not specifically carried out on ‌pregnant women with overweight and obesity.

Recently, several small clinical trials conducted to investigate the effect of myo-inositol supplements on GDM prevention have suggested the effect of myo-inositol on reducing the preterm delivery incidence as a secondary outcome [26–28]. The hypothesis of the effect of myo-inositol on preterm delivery prevention has been proposed by Sharma et al. [24]. According to them, the physiological decrease in uterine-placental inositol levels, in connection with the increasingly proinflammatory placental environment, causes spontaneous rupture of the placental membrane and the onset of labor. Thus, higher uterine-placental inositol levels, potentially increased by maternal administration of myo-inositol supplementation, may reduce eicosanoid production, lipid metabolism, and the secretion of proinflammatory chemocytokines, which generally affect the placental uterine environment responsible for the onset and progression of labor and as a result reduces the preterm delivery risk [24].

In addition, due to the effect of myo-inositol on blood pressure, some evidence suggests that myo-inositol is vital in insulin signaling and improving vascular endothelial function, which can be used as adjunctive therapy in various metabolic diseases such as endothelial disorders and insulin resistance [55, 56]. A recent systematic review showed a significant reduction in systolic and diastolic blood pressure with inositol supplementation [57].

The strength of this research is that it was conducted, for the 1st time, specifically about the effect of myo-inositol on the prevention of GDM in pregnant women with overweight and obesity. One of its limitations is the small sample size. Only one the four clinical trials was specifically conducted on pregnant women with obesity, and three on pregnant women with overweight. Therefore, it is recommended to conduct more studies on pregnant women with overweight and obesity to confirm our findings. On the other hand, three studies were conducted in Italy and only one study was performed in Iran, which had a small sample size and thus reduced the generalizability of our findings. Therefore, the conduction of clinical trials with adequate sample size and power and appropriate quality in different settings is recommended for increasing the generalizability of their results to other communities. Moreover, the conduction of clinical trials on the effect of myo-inositol in combination with a lifestyle change, including diet and physical activities, on pregnant women with overweight and obesity, will be very useful.

## Conclusion

Based on this meta-analysis results, myo-inositol supplementation has shown to be a new and safe preventive strategy in reducing the incidence of gestational diabetes and in regulating FG and 1-h and 2-h OGTT levels, and also in reducing the incidence of GDM complications such as preterm delivery and gestational hypertension in pregnant women with overweight and obesity. However, due to the low quality of the evidence, the conduction of further clinical trials with adequate power and quality is recommended.

## Data Availability

The datasets used or analysed during the current study are available from the corresponding author on reasonable request.

## Abbreviations

GDM: Gestational diabetes mellitus
MI: Myo-inositol
RCT: Randomized controlled trial
FG: Fasting glucose
OGTT: Oral glucose tolerance test
MD: Mean difference
CI: Confidence interval
OR: Odds ratio
T2DM: Type 2 diabetes mellitus
IDF: International Diabetes Federation
PCOS: Polycystic ovarian syndrome
BMI: Body mass index
NICU: Neonatal intensive care unit
RoB: Risk of bias
GRADE: The Grades of Recommendation, Assessment, Development & Evaluation Working Group
MeSH: Medical subject headings
ADA: American diabetes association
ACC: Acetyl-CoA carboxylase
